# Accuracy of point-of-care ultrasound for diagnosis of elbow fractures in children

**DOI:** 10.1186/2036-7902-4-S1-A19

**Published:** 2012-12-18

**Authors:** Joni E Rabiner, H Khine, JR Avner, LM Friedman, JW Tsung

**Affiliations:** 1 Department of Pediatrics, Division of Pediatric Emergency Medicine, Children’s Hospital at Montefiore / Albert Einstein College of Medicine, Bronx, NY, USA; 2 Department of Emergency Medicine, Division of Pediatric Emergency Medicine, New York, NY, USA

## Background

Ultrasound (US) has been shown to be useful in the diagnosis of pediatric skeletal injuries. It can be performed accurately and reliably by emergency department (ED) physicians with focused US training.

## Objective

To determine the test performance characteristics for point-of-care US performed by pediatric emergency medicine (PEM) physicians compared to radiographic diagnosis of elbow fractures and to compare inter-rater agreement between enrolling physicians and an experienced PEM sonologist.

## Patients and methods

This was a prospective study of children up to 21 years old presenting to the emergency department with elbow injuries requiring X-rays. Before obtaining X-rays, PEM physicians performed a focused elbow US. A positive US for fracture at the elbow was defined as the PEM physician’s determination of an elevated posterior fat pad (PFP) and/or lipohemarthrosis (LH) of the PFP. All patients received an elbow X-ray in the ED and clinical follow-up. The gold standard for fracture was fracture on initial or follow-up X-rays.

## Results

One hundred thirty patients with a mean age of 7.5 years were enrolled by 26 sonologists. Forty-three (33%) patients had an X-ray positive for fracture. A positive elbow US had a sensitivity of 98% (95% CI 88-100%), specificity of 70% (95% CI 60-79%), positive likelihood ratio of 3.3 (95% CI 2.4-4.5), and negative likelihood ratio of 0.03 (95% CI 0.01-0.23) for fracture. The inter-rater agreement (kappa) was 0.77. The use of elbow US would reduce X-rays in 48% of patients but would miss 1 fracture.

## Conclusion

Point-of-care US is highly sensitive for elbow fractures, and a negative US may reduce the need for X-rays in children with elbow injuries. Elbow US may be useful in settings where radiography is not readily accessible or time-consuming to obtain.

